# Rapid Construction and Characterization of Infectious cDNA Clones and Reporter Viruses of Enteroviruses, Including Enterovirus A71 and Coxsackievirus B5, with Systematic Identification of Critical Determinants for Successful Reporter Virus Generation

**DOI:** 10.3390/v18050514

**Published:** 2026-04-29

**Authors:** Hao Zheng, Tong Zhao, Meixian Fu, Zirui Niu, Yifan Xing, Xia Cai, Jian-Er Long

**Affiliations:** 1 Key Laboratory of Medical Molecular Virology (MOE/NHC/CAMS), Shanghai Institute of Infectious Disease and Biosecurity, School of Basic Medical Sciences, Shanghai Medical College, Fudan University, Shanghai 200032, China; 2 Department of Medical Microbiology and Parasitology, School of Basic Medical Sciences, Shanghai Medical College, Fudan University, 138 Yixueyuan R., Shanghai 200032, China; 3 Biosafety Level-3 Laboratory, Fudan University, Shanghai 200032, China

**Keywords:** enterovirus A71, coxsackievirus B5, infectious cDNA clone, reporter virus, determinants for reporter virus

## Abstract

Enteroviruses are positive-sense single-stranded RNA viruses and common pathogens that are responsible for diverse public health diseases. To facilitate the study of the virus biology and pathogenesis of enterovirus, we developed a rapid method for construction of the enteroviral cDNA clones including enterovirus A71 (EV-A71) and coxsackievirus B5 (CVB5). As described for EV-A71, the full-length cDNA of CVB5 was amplified by long-distance PCR and cloned into a T7 promoter-containing plasmid using directional seamless cloning technology. The virus was successfully rescued by single transfection into cells stably expressing T7 polymerase and exhibited characteristics similar to the parental virus. Next, through systematic construction and the optimization of the EV-A71 and CVB5 reporter viruses, we successfully generated two novel reporter virus panels with high virus titers, rapid replication, and relatively stable genetic inheritance across passages using the new fluorescence proteins mScarlet3-H and the smallest miRFP670nano3. Analysis of critical determinants for the reporter virus construction revealed that reporter gene sizes, genomic insertion sites, and the usage of protease recognition sites are crucial parameters. The EV-A71 and CVB5 reporter viruses enable antiviral drug evaluation, as demonstrated by our identification of gemcitabine as a broad-spectrum inhibitor of both viruses. These systems also facilitate the functional interrogation of host factors, exemplified by our discovery that METTL3 promotes EV-A71 and CVB5 replication. These reverse genetic tools, including infectious cDNA clones and reporter viruses, will advance basic enterovirus biology and accelerate antiviral drug discovery.

## 1. Introduction

Enterovirus, belonging to the family *Picornaviridae*, is a positive-sense single-stranded RNA virus that is responsible for diverse public health diseases transmitted primarily via the fecal–oral route, respiratory secretions, or direct contact [1]. Within this genus, enterovirus 71 (EV-A71), a member of species A, represents a major causative agent of hand, foot, and mouth disease in children and exhibits pronounced neurotropism, frequently causing severe neurological complications including aseptic meningitis, brainstem encephalitis, and fatal neurogenic pulmonary edema [2,3,4]. Coxsackievirus B5 (CVB5) is a member of the Enterovirus species B in the family *Picornaviridae*. Infection with Enterovirus B (including types 1 to 6) is usually asymptomatic or mild but may occasionally result in severe diseases of the heart, pancreas, and central nervous system. Clinical manifestations include aseptic meningitis, encephalitis acute flaccid paralysis, myocarditis, Type 1 diabetes, herpangina, and hand, foot, and mouth disease (HFMD) [5,6,7,8,9,10]. Similar to EV-A71, CVB5 has also been reported in Asia [11,12,13], Europe [14], and North America [15]. Despite the substantial disease burden posed by these multitype, highly pathogenic enteroviruses, no approved broad-spectrum antiviral drugs are currently clinically available, and the existing vaccines fail to provide cross-serotype protection. Therefore, investigating the pathogenic mechanisms of enteroviruses such as EV71 and CVB5, and identifying safe and effective broad-spectrum antivirals, represent critical unmet needs [16].

Enterovirus is a nonenveloped virus with a single-stranded positive RNA genome of approximately 7.4 kb in length, which includes a major open reading frame (mORF) between 5′-UTR and 3′-UTR. The viral mORF encodes a large polyprotein that is subsequently cleaved by viral proteases 2A^pro^ and 3C^pro^ into three precursor proteins, designated P1, P2, and P3. P1 is further processed to yield four structural proteins (VP1, VP2, VP3, and VP4) that constitute the viral capsid. P2 is processed into 2A^pro^, 2B, and 2C, while P3 is cleaved into 3A, 3B (VPg), 3C^pro^, and 3D^pol^ [17]. Both 2A^pro^ and 3C^pro^ are cysteine proteinases. 2A^pro^ autocatalytically processes its N-terminus, separating the P1 precursor proteins from the nonstructural P2 and P3 polyproteins. Subsequent cleavage events are primarily mediated by 3C^pro^ or its precursor 3CD^pro^, with the exception of VP4 and VP2, which are generated by VP0 autolysis during progeny virus assembly [18,19]. The viral proteases played an important role in causing cell death, inhibiting the host antiviral response, and resulting in host diseases [20]. However, the molecular mechanisms for the pathogenesis are largely unknown. Infectious clone is a common reverse genetic tool for studying the virus functional genomics. It would benefit the development of vaccines and antiviral drugs against the RNA viruses. Many infectious cDNA clones of enteroviruses have been successfully constructed, including our and other groups’ described EV-A71 [21,22,23], CA6 [24], CA10 [25], CA16 [24], ECHO25 [26], and ECHO30 [27]. Up to now, for the infectious clone of CVB5, only two clinical strains have been reported [28,29,30]. It is necessary to develop a rapid method and construct a new virus strain of CVB5 to facilitate the understanding of the evolution of the virus, as well as the development of CVB5 vaccines and the specific antiviral drugs.

Reporter viruses are recombinant viruses generated through reverse genetics by inserting exogenous tracer tags—such as fluorescent proteins (GFP, RFP) or luciferases—at specific genomic sites [31,32]. Compared with traditional endpoint assays relying on cytopathic effect (CPE) observation, plaque assays, or cell viability detection, reporter virus systems enable real-time, non-invasive, and dynamic tracking of the entire viral life cycle—including entry, genome replication, protein expression, and progeny release—without disrupting the host cells, by converting invisible viral propagation into quantifiable optical signals. Leveraging this core advantage, reporter viruses have been extensively applied in large-scale high-throughput antiviral drug screening, neutralizing antibody titer evaluation, and the mechanistic dissection of virus–host interactions [31,33,34]. However, constructing efficient and stable enterovirus reporter viruses remains technically challenging. First, the icosahedral capsid of enteroviruses imposes strict physical packaging limits; the insertion of large exogenous reporter genes (e.g., EGFP or firefly luciferase) frequently prevents effective nucleocapsid assembly, resulting in extremely low rescue titers or complete rescue failure [35,36]. Second, exogenous gene introduction imposes an additional replicative burden on the virus; to restore fitness and replicative adaptation, recombinant enteroviruses readily undergo homologous recombination and spontaneous exogenous sequence deletion during serial passage, exhibiting marked genetic instability [37,38]. Furthermore, the existing fluorescent enterovirus reporter systems applied to microplate-based high-throughput screening suffer from reduced sensitivity and signal-to-noise ratios due to interference from phenol red in the culture medium and cellular autofluorescence [30]. These technical bottlenecks severely constrain the broader application of reporter viruses in enterovirus biological research and clinical pharmacodynamic evaluation.

In this study, we reported a rapid method to construct a novel cDNA clone of CVB5, as previously described for that of EV-A71. The viruses were successfully rescued by a single transfection of a cDNA clone into a cell line stably expressing T7 polymerase, showing similar characteristics in comparison to the parent virus. Based on these cDNA clones, we further performed the construction and optimization of EV-A71 and CVB5 reporter viruses, which generated two novel reporter virus panels with the novel fluorescence protein mScarlet3-H and the smallest miRFP670nano3. An analysis of the critical determinants for enterovirus reporter virus construction revealed that reporter gene size, genomic insertion site, and the protease recognition site for reporter release are crucial parameters. These reporter viruses facilitate antiviral screening and basic virological research, as demonstrated by our identification of gemcitabine as a broad-spectrum inhibitor of both viruses and METTL3 as a proviral host factor promoting EV-A71 and CVB5 replication.

## 2. Materials and Methods

### 2.1. Virus Strain, Cell Lines, and Reagents

The EV71 infectious cDNA clone and the cells stably expressing the T7 polymerase gene (293FT-T7pol) were as previously described [23]. The clinical CVB5 strain SHA/CHN/1984 (GenBank Accession No. ON741190) was utilized to construct a cDNA clone and further for reporter virus construction. Rhabdomyosarcoma (RD), HEK293FT, Vero, and HeLa cell lines were cultured in high glucose DMEM supplemented with 10% fetal bovine serum (FBS). Mouse polyclonal antibodies against CVB5 (MAB9410) were purchased from EMD Millipore Corporation.

### 2.2. Phylogenetic Analysis and Boot Scanning Assay

MEGA X (v10.2.6) was used for phylogenetic tree construction using the maximum likelihood analysis as previously described [39]. Briefly, the sequence alignments were performed by Clustal W, with the Kimura-2 parameter as a model for nucleotide substitution. The default parameter values and settings were utilized as recommended in the software. The robustness of the tree was determined by bootstrapping with the usage of 1000 pseudo replicates. Simplot v3.5.1 (https://sray.med.som.jhmi.edu/SCRoftware/SimPlot/, accessed on 29 March 2026) was further used for boot scanning and recombination analysis of CVB5 strains as described in [40].

### 2.3. Construction of CVB5 cDNA Clone

For rapid construction of the CVB5 cDNA clone, the strain SHA/CHN/1984 was applied to infect HeLa cells at an MOI of 10 for 16 h, then the total RNA was extracted with the TRIzol reagent. The viral RNA was reverse transcribed to cDNA by the SuperScript^TM^ II Reverse Transcriptase (Invitrogen, Carlsbad, CA, USA) with a primer including oligo (dT)_30_ (5′-ggatctacgcgt_30_ccg-3′) after the removal of genomic DNA using a PrimeScript™ RT Reagent Kit with gDNA Eraser (Takara, Kusatsu, Shiga, Japan). The full-length genome of CVB5 was amplified by a touchdown long-distance PCR using iProof High-Fidelity DNA Polymerase (Bio-Rad Laboratories, Hercules, CA, USA). The forward primer included a T7 promoter, as previously reported [23], and the reverse transcribed primer was also utilized as the reverse primer. The PCR amplification was performed by the touchdown program (denatured at 98 °C for 30 s, followed by two cycles of annealing at 46 °C and 10 cycles of annealing at 68–63.5 °C (−0.5 °C/cycle), and continued 15 cycles of annealing at 63 °C, and finally incubated at 72 °C for 7 min). The expected PCR products were purified and amplified again by a new pair of primers (forward primer: 5′-atgtacgggccagatatacgcgtggtaatacgactcactatagggtta-3′; reverse primer: 5′-ggtttaaacgggccctctagaggatctacgcgt_30_ccg-3′). The full length of the CVB5 genome was then cloned by the Kit NovoRec^®^ plus One step PCR Cloning (Novoprotein Scientific Inc., Shanghai, China) into the plasmid of pcDNA3.1, in which the CMV promoter was previously deleted through the endonucleases of *Mlu* I and *Xba* I (Takara, Kusatsu, Shiga, Japan). The infectious clone was identified by the endonucleases and confirmed by the sequencing analysis.

### 2.4. Rescue of Infectious Virus and Plaque Assay

The cDNA clone of CVB5 (pcDNA3.1-ΔCMV-T7-CVB5) was transfected into the cells of 293FT-T7pol at 4.0 μg/well in a six-well plate with lipofectamine 2000. The culture supernatant was collected as an infectious virus at 72 h post-transfection. The virus titer was quantified by plaque assay as in our previous description [41]. Briefly, Vero cells were seeded at 5 × 10^5^ cells/well in a six-well plate and infected with 0.5 mL of the serially diluted virus. After discarding the virus solution, the cells were covered with culture media including 0.5% low-melt agarose for another 60 h. The plaques were stained with 0.1% crystal violet for the quantification of the virus titers.

### 2.5. Cell Morphology and Microscopy of Rescued Virus-Infected Cells

Vero and HeLa cells were seeded and infected with the indicated virus (MOI = 10 PFU/cell) for observation under microscopy to identify the morphological characteristics of virus-infected cells. The cells were also infected with the recued virus at an MOI of 10 and fixed with 4% formaldehyde at 8 h post-infection (hpi). The fixed cells were sequentially stained with the polyclonal antibodies against CVB5, the second antibodies (goat anti-mouse IgG-Dye-light 594), and DAPI. The images were captured with an EVOS F1 microscope (Advanced Microscopy Group, Bothell, WA, USA).

### 2.6. Rescued-Virus Growth Curve Detection

HeLa cells were seeded at 1 × 10^6^/flask in a T25 flask for 16 h. The rescued and the parent virus were respectively applied to the cells at an MOI of 10 PFU/cells. The infected cells were collected at the indicated time points to quantify the copies of viral RNA genome by standard quantitative RT-PCR (qPCR), and the culture supernatant was applied to quantify the virus titer by plaque assay as described above.

### 2.7. Construction and Characteristics of EV-A71 and CVB5 Reporter Virus

We initially inserted GFP cDNA between the VP1 and 2A genes of EV71 and CVB5 infectious clones to generate pcDNA3.1-EV71-GFP-2A and pcDNA3.1-CVB5-GFP-2A. To ensure the correct post-translational cleavage of the viral polyprotein and minimize packaging interference from the inserted reporter gene, we flanked the GFP gene with their endogenous 2A^pro^ recognition sequences (TTL↓GKF for EV-A71; QTT↓GAF for CVB5), thereby enabling self-cleavage-mediated separation of the exogenous reporter protein from viral structural proteins through the viral 2A protease mechanism. These constructs were transfected into 293FT/T7pol cells. Transfected cells were freeze-thawed to release the intracellular virus, which was then used to infect the RD cells. GFP expression in transfected and infected cells was monitored by fluorescence microscopy to confirm successful reporter virus rescue. Following initial validation, we characterized plaque morphology, quantified viral replication by flow cytometry, and generated growth curves. Serial passaging was performed to assess the genetic stability, with GFP retention monitored by RT-PCR and semi-quantitative PCR.

### 2.8. Systematic Optimization of Reporter Virus Construction

To identify better genomic insertion sites for the reporter, we inserted GFP at multiple genomic positions based on viral polyprotein processing patterns, then systematically evaluated the rescue efficiency and reporter tolerance at each site according to a uniform experimental flowchart. Briefly, the genomic sites for the inserted reporter included 5′-UTR/VP4, VP1/2A (P1/P2 junction), 2A/2B, 2C/3A (P2/P3 junction), and 3D/3′-UTR, using the indicated cleavage sites (Figure S1A). Each design for a candidate reporter virus underwent plasmid construction and purification, then the candidate clone was transfected into 293FT/T7pol cells at 4 μg/well in a 6-well plate. The transfected cells were collected and underwent three freeze–thaw cycles to release the progeny virus. The cell lysate was centrifuged and further clarified through the filter of 0.45 μm membrane. The solution was utilized to infect the susceptible cell lines: specifically, RD for EV-A71, and HeLa for CVB5 reporter virus infection. The expressions of fluorescence were observed under a microscope or by FACS assay to evaluate the infectivity and efficiency of the reporter virus (Figure S1B). For the fast-qualitative screening, each clone of the reporter virus was tested by at least three independent experiments of transfection and infection.

To identify better fluorescent genes for the reporter virus, for promising insertion sites, we utilized mScarlet3-H (an extremely stable monomeric RFP) and miRFP670nano (the smallest near-infrared fluorescence available) to displace the GFP, and further assessed the efficiency of different reporters amongst the rescued reporter viruses. Additionally, to identify suitable protease recognition sites for the reporter release by virus, we also evaluated the protease cleavage site configurations, while the protease recognition sites were flanked to the reporters, comparing the 2A protease and 3C protease recognition sites, as well as variations in site length and sequence integrity. The evaluations for the reporter virus designs referred to above also underwent the uniform experimental flowchart (Figure S1B). Through these systematic comparisons, we collected multiple optimized EV-A71 and CVB5 reporter viruses and further characterized their plaque formation, replication kinetics, and genetic stability.

### 2.9. Activity of the Candidate Drugs Against Enterovirus and Evaluated by the Reporter Viruses

To validate the reporter viruses for antiviral screening, we selected two candidate compounds—lotamilast and gemcitabine—identified from an FDA-approved pediatric clinical drug library . We first determined their CC_50_, EC_50_, and selectivity index (SI) against EV-A71, as previously described [42]. Briefly, RD cells were seeded in 96-well plates for 12 h. Then, the cells were treated by the drugs starting at 20 μM and serially diluted by 2- or 3-fold, and simultaneously infected with EV-A71at an MOI of 0.5 for another 72 h. The cell viability was detected with a CCK-8 kit, according to the producer’s instructions. The values of CC_50_ and EC_50_ were counted by fitting the standard curves of dose–response. The two selected candidates were subsequently evaluated using reporter viruses for further evaluation. EV71-mScarlet3-2A and CVB5-miRFP670nano3-2A were applied to infected RD and Vero cells, respectively, and treated or not by the drugs at 1.0 μM. The cell morphology and fluorescence were observed under a microscope, or the fluorescent-positive cells were quantified by FACS at the indicated time points post-infection.

### 2.10. Generation of METTL3-Knockout Cells and Infected by Reporter Viruses

To further validate the utility of these reporter viruses for investigating the host factor function, we selected METTL3 as a test case. METTL3-knockout (KO) COS-7 and RD cells were generated by a CRISPR-Cas9 system, using the gRNAs as previously described [41]. Briefly, gRNA1 (5′-gagttgattgaggtaaagcg-3′) and gRNA2 (5′-atgttaaggccagatcagag-3′) were designed to target METTL3. LentiCRISPRv2 plasmid (Addgene) expressing Cas9 and guide RNA was co-transfected with packaging plasmids to produce infectious lentiviruses, which were collected to infect COS-7 and RD cells. Cells were selected with puromycin and the knockdown efficiency was assessed by Western blot, using polyclonal antibodies (Proteintech, Rosemont, IL, USA). Cells transduced with control LentiCRISPRv2 plasmid served as negative controls. METTL3-knockout RD cells were infected with EV71-miRFP670nano3-2A reporter virus, and METTL3-knockout COS-7 cells with CVB5-miRFP670nano3-2A, both at MOI = 0.1. The infected cells were harvested at the indicated time points for flow cytometric analysis.

### 2.11. Statistical Analysis

Data were analyzed using the software of GraphPad Prism v10.0. Growth curves were analyzed using two-way ANOVA with multiple comparisons. Pairwise comparisons between two groups were performed using unpaired Student’s *t*-tests. Statistical significance was defined as *p* < 0.05.

## 3. Results

### 3.1. Rapid Construction of Infectious cDNA Clone of CVB5

**Figure 1:**
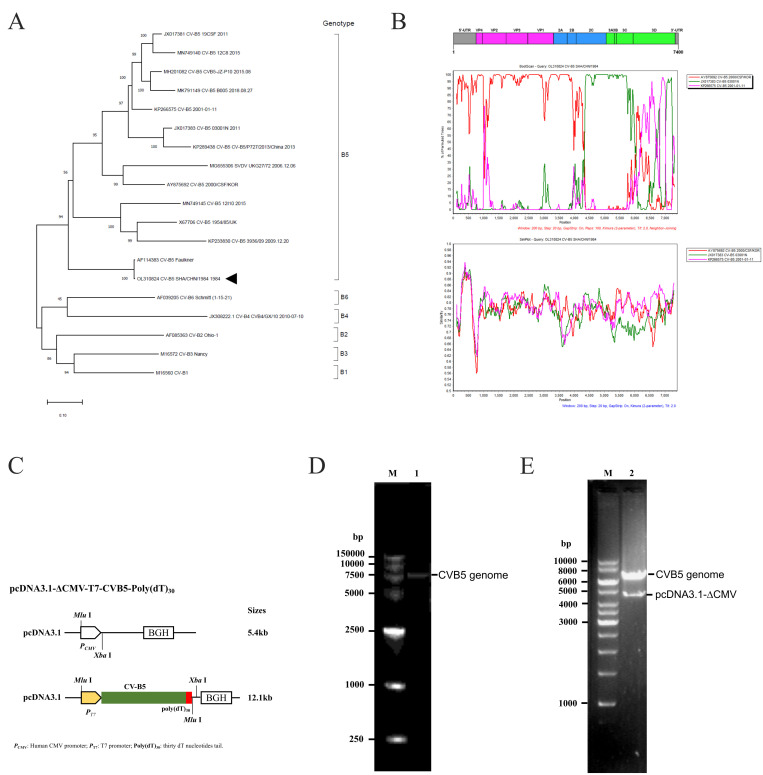
Construction of the infectious cDNA clone of CVB5 using the clinical strain SHA/CHN/1984. (**A**) Phylogenetic analysis of the clinical CVB5 strains on the full-length genome. Phylogenetic trees were constructed using the maximum likelihood method by MEGA X (v10.2.6). The robustness of the tree was determined by bootstrapping by using 1000 pseudo-replicates. The scale bar denotes a measurement of relative phylogenetic distance, and the triangle marks the cloned strain in this study. (**B**) Boot scanning and similarity plot analyses based on the full-length genomes of CVB5 in comparison with other CVB5 strains. (**C**) Strategy of cloning of the full-length genome of CVB5. The CMV promotor in the pcDNA3.1 plasmid was removed by *Mul* I and *Xba* I, and then the genome of CVB5 flanked with a poly(dT)30 at 3 terminals was cloned using Gibson assembly with the two restriction sites. (**D**) One-step long-distance PCR for amplifying the full length of the CVB5 genome. The cDNA of the CVB5 genome with a poly(dT)_30_ is about 7.4 kb in Lane 1. (**E**) The cDNA clone of CVB5 was identified by the endonucleases. The plasmid of pcDNA3.1 with the CMV-promotor deletion is about 4.7 kb in Lane 2.

For the rapid construction of the infectious cDNA clone of CVB5, a clinical strain SHA/CHN/1984 of CVB5 that was replicated efficiently in HeLa and Vero cells was selected. Based on the results of the phylogenetic analysis and Bootscan (Figure 1A), this virus strain was on the same branch as the prototype strain Faulkner when the phylogenetic tree was constructed using the full-length genome sequences by the maximum likelihood method [43]. Further recombination analysis with Simplot [44] showed that in the VP4-VP1, 2A-2B region (located at 1300–4200 in the genome), the virus strain SHA/CHN/1984 had high support values (more than 90%) for the recombination of the CVB5/2000/CSF/KOR (AY875692) (Figure 1B). In the region of partial 2C to 3C (located at 4300–5900 in the genome), the virus strain SHA/CHN/1984 showed the high supportive value of the recombination of the strain 03001N (JX017383). The two strains, CVB5/2000/CSF/KOR (AY875692) and 03001N (JX017383) were isolated in Korea in 2000 and in Jinan of Shandong Province in 2003, indicating that the two strains could stand a chance to be originated from the strain SHA/CHN/1984, given the time and the location of their isolation. The X-type intersection occurs in part of the 2C region (4200–4300), suggesting a possible recombination breakpoint (Figure 1B).

For rapid construction of the cDNA clone using a new CVB5 strain, the strain SHA/CHN/1984 was applied to infect HeLa cells, then the viral RNA in the cells was extracted and further reverse-transcribed to cDNA. The full-length genome of CVB5 was amplified by a touchdown long-distance PCR using a high-fidelity DNA polymerase (Figure 1D). The full length of the CVB5 genome was then cloned using the seamless cloning technology into the plasmid of pcDNA3.1, in which the CMV promoter was previously deleted through the endonucleases of *Mlu* I and *Xba* I (Figure 1C). The infectious clone was identified by the endonucleases (Figure 1E) and confirmed by the sequencing analysis. The results showed that the mutation was not observed by the PCR amplification. In comparison with the previous cloned prototype strain Faulkner, our infectious clone of CVB5 included 20-nucleotide differences, resulting in three amino acid mutations. One mutation was located at 4185 a > g in the 2C gene and resulted in the amino acid mutation of Asn to Ser, and the other two amino acid mutations were located at 5282 t>a in the 3A gene (Phe > Ile), and 6912 c > t in the 3D gene (Ser > Phe), respectively.

### 3.2. Characteristics of Infectious cDNA Clone of CVB5

**Figure 2:**
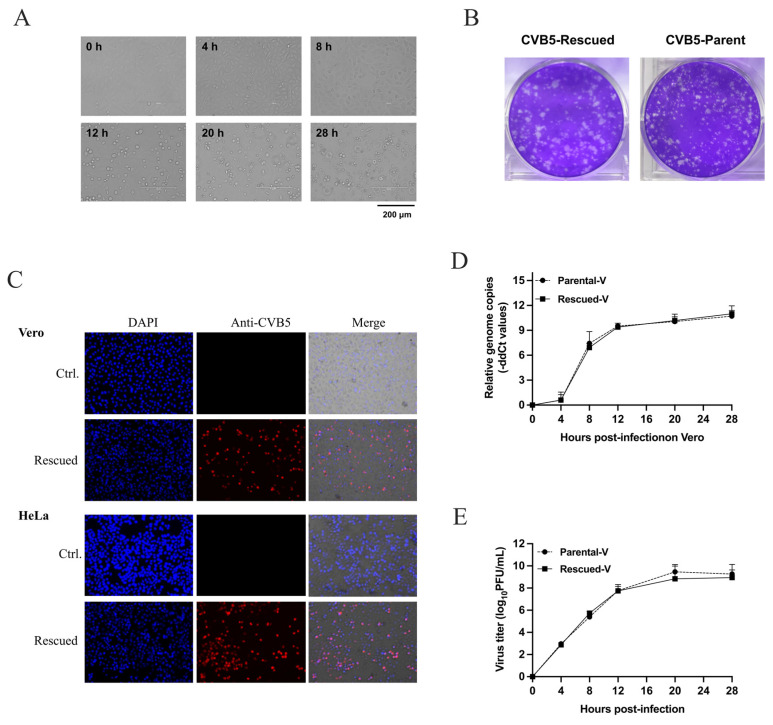
Characteristics of the rescued CVB5. (**A**) Rescued virus induced CPEs on Vero cells. The cells were observed at the indicated time points when infected with an MOI of 10. (**B**) Plaques formed on Vero cells when infected with an equal amount of rescued and parent virus. (**C**) Rescued virus infection was detected with polyclonal antibodies against CVB5. Images were captured through microscopy at 16 hpi when Vero and HeLa cells were infected at MOI = 10. (**D**) The rescued and the parent virus showed a similar RNA replication curve on the Vero cells. The cells were infected with the rescued and parent virus at MOI = 10. The infected cells and the culture supernatant were collected at the indicated time points. The viral RNA genome in the infected cells was detected by quantitative PCR with β-actin as an internal control. Data were normalized by β-actin and the cut-off value of Ct in the original time point. (**E**) Plaque assay of the virus in the culture supernatant of Vero cells. Cells were infected as panel (**D**). The data are presented as geometric mean ± SD from three independent experiments. Comparisons between two groups were performed using two-way ANOVA; differences were not statistically significant (*p* > 0.05).

To rapidly and efficiently rescue the infectious virus, we transfected the cDNA clone of CVB5 to a stable T7 polymerase-expressing 293HEK/FT cell (293FT/T7pol) as we previously described [23]. Then, the supernatant at 72 h post-transfection was collected as the rescued virus. The rescue efficiency was quantified by plaque assay, yielding titers of 9.8 × 10^9^ PFU/mL from single-plasmid transfection, with subsequent validation by in vitro characterization. After the Vero cells were infected with the rescued virus at an MOI of 10, the images were captured at the indicated time points. The infected cells showed the distinct enterovirus-induced CPEs (Figure 2A), and their cell morphology was found to be similar to the parental virus. The rescued virus formed the similar morphology of plaques on Vero cells to their parent virus (Figure 2B). Upon using the specific antibodies against CVB5, the rescued virus-infected Vero and HeLa cells were both stained with significant signals (Figure 2C). The growth curve of the rescued virus was detected using Vero cells and was found to be similar to that of the parent virus in terms of RNA genomes (Figure 2D) and virus titer (Figure 2E). These results suggested that the rescued virus had similar characteristics to its parent virus.

### 3.3. Construction of GFP-Tagged EV-A71 and CVB5 Reporter Viruses

**Figure 3:**
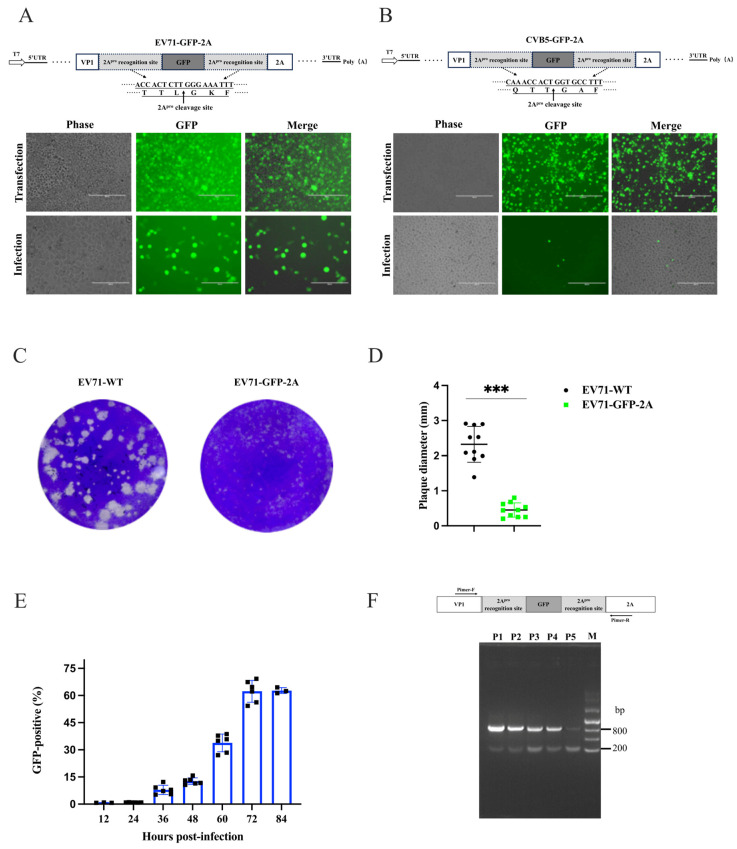
Construction and characteristics of GFP-tagged EV-A71 and CVB5 reporter virus. (**A**) Construction of GFP-tagged EV-A71 reporter virus. Schematic of EV-A71 infectious clone of pcDNA3.1-EV71-2A for reporter virus (**upper**). The GFP reporter gene was inserted at the P1/P2 junction (between VP1 and 2A), flanked by 2A^pro^ recognition sequences (TTL↓GKF) that mediate the self-cleavage and release of the mature fluorescent protein following expression. GFP expressions in the transfected 293FT/T7pol cells at 24 h post-transfection (**middle**), and in RD cells at 48 h post-infection with the transfected cell lysate (**lower**). (**B**) Schematic of CVB5 infectious clone pcDNA3.1-CVB5-2A. The GFP reporter gene was also inserted between VP1 and 2A, flanked by 2A^pro^ recognition sequences (QTT↓GAF). GFP expressions at 24 h post-transfection in the transfected 293FT-T7pol cells (**middle**) and at 48 h post-infection (**Lower**). Scale bar: 400 μm for panel B; 200 μm for panel A. (**C**) Plaques formed by EV71-WT and EV71-GFP-2A in infected cells. (**D**) Quantitative analysis of plaque diameters. Ten well-defined plaques per group were randomly selected for statistical analysis. The pixel ratios were converted to millimeters based on the 35 mm 6-well plate diameter; unpaired Student’s *t*-test, *** *p* < 0.001. (**E**) Growth of EV71-GFP-2A reporter virus in RD cells infected at MOI = 0.1. (**F**) Genetic stability analysis of EV71-GFP-2A reporter virus across passages P1–P5. Schematic of primer locations for PCR validation, using pcDNA3.1-EV71-GFP-2A infectious clone as template (**upper**). Expected PCR product: 876 bp with intact GFP; 102 bp following GFP loss.

To construct GFP-tagged EV-A71 and CVB5 reporter viruses, we first inserted GFP cDNA between the VP1 and 2A genes of EV-A71 and CVB5 infectious clones to generate pcDNA3.1-EV71-GFP-2A and pcDNA3.1-CVB5-GFP-2A, respectively. These constructs were transfected into 293FT/T7pol cells. The transfected cells exhibited robust GFP expression and characteristic cytopathic effects (CPE) in GFP-positive cells, indicating successful viral protein and reporter gene expression (Figure 3A). An intracellular virus was released by freeze–thaw lysis and used to infect cells. EV71-GFP-2A yielded abundant GFP-positive RD cells, whereas CVB5-GFP-2A produced only sporadic, unstable fluorescent signals, demonstrating that pcDNA3.1-EV71-GFP-2A stably generates an infectious EV71-GFP-2A reporter virus, while CVB5-GFP-2A construction requires further optimization (Figure 3B).

The plaque assay showed that EV71-GFP-2A was replicated in RD cells but formed fuzzy plaques and was significantly smaller than that of wild-type EV71 (Figure 3C,D), indicating that GFP insertion severely impairs viral replication and the cell lysis capability. Viral growth analysis revealed that it needs 72 h to reach the high-level plateau (Figure 3E), delaying the replication kinetics in comparison to the wild-type virus [41,45]. Serial passage and RT-PCR/PCR analysis demonstrated progressive reporter gene loss, with reporter virus proportions declining and wild-type EV71 increasing; by passage 5, the reporter gene was essentially lost, indicating the poor genetic stability of EV71-GFP-2A (Figure 3F).

### 3.4. Viral Genomic VP1/2A and 5′-UTR/VP4 Sites Partially Tolerant for Reporter Insertion

**Figure 4:**
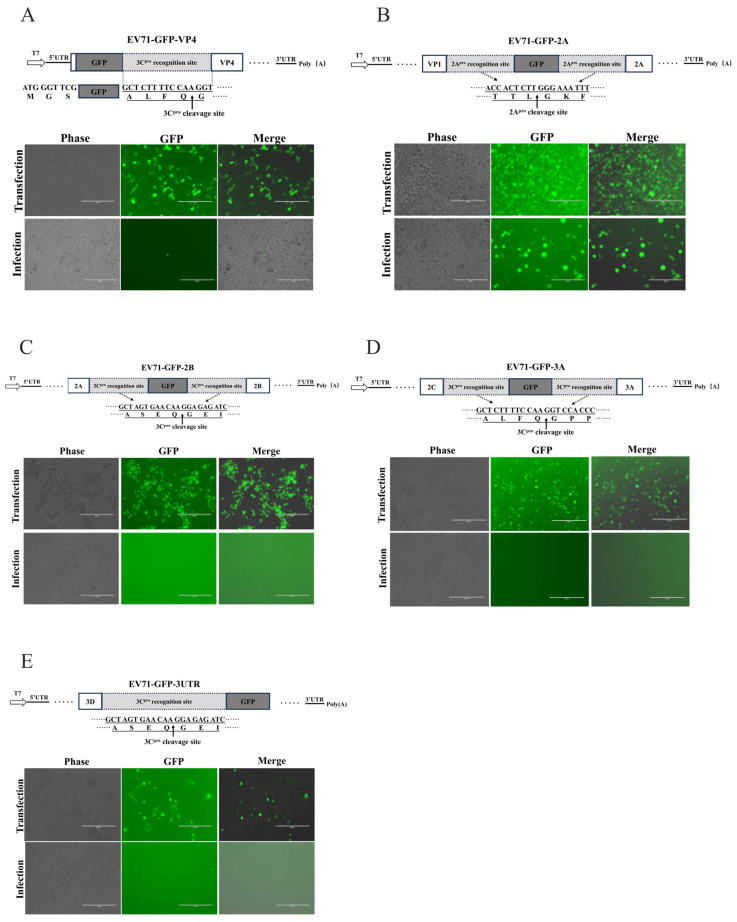
Construction and rescue evaluation of EV-A71 reporter viruses with GFP inserted at distinct sites. (**A**–**E**) illustrate the construction design and rescue outcomes for GFP reporter gene insertion at five distinct genomic sites in the EV71 genome: 5′-UTR/VP4 (**A**), VP1/2A (**B**), 2A/2B (**C**), 2C/3A (**D**), and 3D/3′-UTR (**E**). Schematic diagrams (**upper panels**) detail GFP insertion configurations and the specific protease (3C^pro^ or 2A^pro^) recognition and cleavage sequences introduced. The rescue results show the plasmid transfection (Transfection) and viral infection (Infection) outcomes. Corresponding recombinant infectious clone plasmids were transfected into 293FT-T7pol cells, with fluorescence images captured at 24 h post-transfection. Transfected cells were subsequently harvested, lysed, filtered, and inoculated onto permissive target cells to assess the generation of infectious reporter virus at 48 hpi. Scale bars: 200 μm.

The above results demonstrated that GFP insertion between VP1 and 2A in the EV-A71 genome yielded an infectious progeny virus, whereas an identical strategy almost failed to generate an infectious reporter virus for CVB5. It prompted us to test other alternative insertion sites within the viral genome. Consequently, we generated GFP-containing infectious clones with insertions at multiple genomic sites: 5′-UTR/VP4, VP1/2A (P1/P2 junction), 2A/2B, 2C/3A (P2/P3 junction), and 3D/3′-UTR. At the two ends of GFP in each site, the endogenous protease cleavage sequences were kept based on the viral polyprotein processing patterns, except the 5′-UTR/VP4 and 3D/3′-UTR sites; only the end of the GFP, connected with viral proteins, was supplemented with 3C^pro^ recognition sites (Figure 4A–E). For fast qualitative screening of the productive reporter virus, we performed the uniform experimental flowchart and tested it on at least three cycles of transfection and infection (Figure S1). The results exhibited that all of the sites with inserted GFP observed GFP expression, although the GFP levels and distribution were slightly different (Figure 4A–E). However, only the VP1/2A site in EV-A71 yielded efficient reporter virus rescue (Figure 4B), and the 5′-UTR/VP4 site produced minimal rescue (Figure 4A). Although we did not quantify the reporter virus titers, these data indicate that the insertion site modestly affects viral gene expression but substantially impairs progeny virion packaging. Similar results were also observed in CVB5, and all sites failed in the CVB5-reporter, except for the VP1/2A site at a very low efficiency (Figure S2A–D).

### 3.5. Fluorescence Reporter Gene Sizes Significantly Affected the Efficiency of Rescuing Report Virus

**Figure 5:**
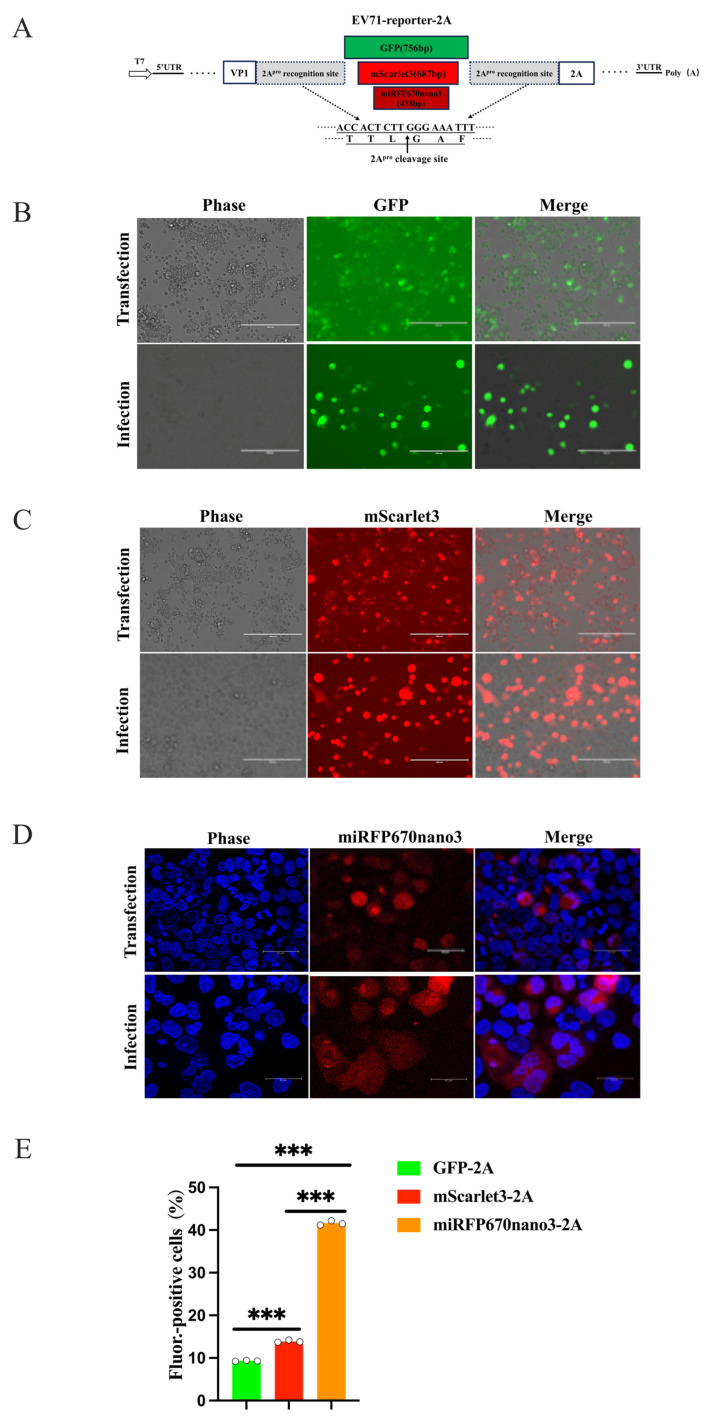
Novel fluorescence proteins exhibited advantages over GFP in the rescue of EV-A71 reporter virus. (**A**) Schematic of reporter virus construction strategy showing novel reporter gene insertion between VP1 and 2A in the EV-A71 genome. (**B**–**D**) Rescue and infection validation of pcDNA3.1-EV71-GFP-2A (**B**), pcDNA3.1-EV71-mScarlet3-2A (**C**), and pcDNA3.1-EV71-miRFP670nano3-2A (**D**) infectious clones. (**E**) Quantitative rescue efficiency determined by flow cytometric analysis of fluorescent cell percentages. Data represent mean ± SD from three independent replicates; unpaired Student’s *t*-test, *** *p* < 0.001. Scale bars: 200 μm for (**A**–**C**) and 40 μm for (**D**).

The VP1/2A site demonstrated clear advantages for EV71-GFP reporter virus rescue; however, CVB5-GFP rescue remained unsuccessful (Figure 4 and Figure S2), suggesting that the recombinant genome exceeded the capsid packaging capacity, and this indicated that different enteroviruses exhibit distinct tolerance limits for foreign genetic material, with CVB5 possibly possessing a narrower genomic capacity than EV-A71. We therefore introduced smaller reporter genes—mScarlet3-H and the substantially smaller far-red protein miRFP670nano3—under the optimized strategy (VP1/2A site) (Figure 5A). The results showed that substitution with novel fluorescent reporter genes substantially improved the rescue efficiency (Figure 5 and Figure S3). The EV71-mScarlet3-2A reporter virus exhibited significantly brighter fluorescence than GFP (Figure 5B,C). EV71-miRFP670nano3-2A, carrying a near-infrared fluorescent protein, was visualized by confocal microscopy at 633 nm, revealing abundant fluorescent cells (Figure 5D). The flow cytometric quantification of the rescue efficiency demonstrated that recombinant infectious clones carrying novel fluorescent proteins achieved significantly higher rescue rates than traditional GFP-tagged constructs (Figure 5E).

Given the extreme instability of GFP-tagged CVB5 reporter viruses and leveraging the successful optimization experience from EV-A71, we constructed pcDNA3.1-CVB5-mScarlet3-2A and pcDNA3.1-CVB5-miRFP670nano3-2A infectious clones based on the previous pcDNA3.1-CVB5-GFP-2A backbone (Figure S3A,B). Remarkably, reporter gene substitution alone enabled the successful rescue of highly infectious reporter viruses (Figure S3C,D). These results demonstrate that reporter gene size is critical for rescue success, and this modification not only improved the EV-A71 rescue efficiency but also enabled the successful generation of CVB5 reporter viruses carrying mScarlet3-H and miRFP670nano3.

### 3.6. Protease Recognition Sites and Their Length Altered the Efficiency of Rescuing Reporter Virus

**Figure 6:**
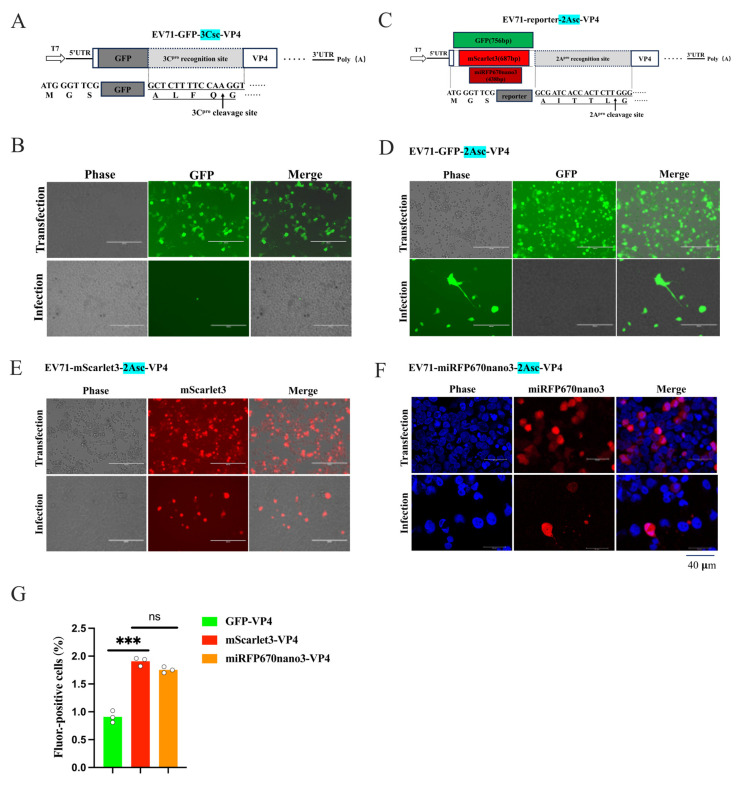
Viral protease cleavage strategy for reporter release significantly impacted the efficiency of rescuing EV-A71 reporter virus. Optimization of protease cleavage strategy (3C^pro^→2A^pro^) and rescue efficiency evaluation at the EV71 5′-UTR/VP4 site. (**A**) Schematic of reporter virus construction strategy of pcDNA3.1-EV71-GFP-3Csc-VP4 infectious clone, which was transfected into 293FT-T7pol cells and cell lysate used to infect RD cells. (**B**) Replacement of 3C protease recognition sequence with 2A protease recognition sequence, generating pcDNA3.1-EV71-GFP-2Asc-VP4. (**D**,**E**) Substitution with novel fluorescent reporters mScarlet3-H (**D**) and miRFP670nano3 (**E**) using optimized 2A^pro^ cleavage strategy. Confocal microscopy with DAPI-stained nuclei (blue) (**F**). (**G**) Flow cytometric quantification of rescue efficiency using different reporter genes at 5′-UTR/VP4 site. Data represent mean ± SD from three independent replicates; unpaired Student’s *t*-test, *** *p* < 0.001; and “ns” indicates no signifieant differenee.

It was reported that the reporter enteroviruses were successfully rescued with the 5′-UTR/VP4 site to insert reporters [30,46]. However, at the 5′-UTR/VP4 site, the pcDNA3.1-EV71-GFP-VP4 infectious clone employing the 3C protease cleavage strategy yielded suspected reporter virus rescue at an extremely low efficiency (Figure 5A). To improve the low rescue efficiency at the 5′-UTR/VP4 site in EV71, we optimized protease recognition sites for the reporter cleavage. Replacement of the 3C protease site (3Csc) with the 2A protease site (2Asc) yielded pcDNA3.1-EV71-GFP-2Asc-VP4 and pcDNA3.1-EV71-GFP-3Csc-VP4 (Figure 6A,B). Remarkably, substitution with the 2A^pro^ strategy yielded abundant bright green fluorescence in the target cells (Figure 6B), confirming that 2A^pro^ efficiently mediates polyprotein cleavage and viral rescue at this site.

Having established the superiority of 2A^pro^ over 3C^pro^ at the 5′-UTR/VP4 site, we sought to further improve the rescue efficiency by replacing GFP with the smaller fluorescent tags mScarlet3-H and miRFP670nano3. Transfection and infection validation demonstrated a successful rescue of both novel recombinant viruses, with robust red or near-infrared fluorescence in host cells (Figure 6E,F). Flow cytometric analysis of early infection revealed that, under identical 5′-UTR/VP4 insertion and 2A^pro^ cleavage conditions, recombinant viruses carrying mScarlet3-H or miRFP670nano3 achieved significantly higher rescue efficiencies than the traditional GFP construct (Figure 6G). These findings further validate that appropriate protease site selection at the 5′-UTR/VP4 position, combined with reduced reporter gene size, substantially enhances the reporter virus rescue efficiency. For CVB5, simultaneous replacement of the protease site (3Csc→2Asc) and reporter gene (GFP→miRFP670nano3) enabled successful generation of CVB5-miRFP670nano3-VP4, although the rescue efficiency was lower than that in EV-A71 (Figure S4 and Figure 6F). These findings further confirmed that 2Asc outperforms 3Csc at the 5′-UTR/VP4 site, and smaller reporter genes facilitate a successful rescue.

**Figure 7:**
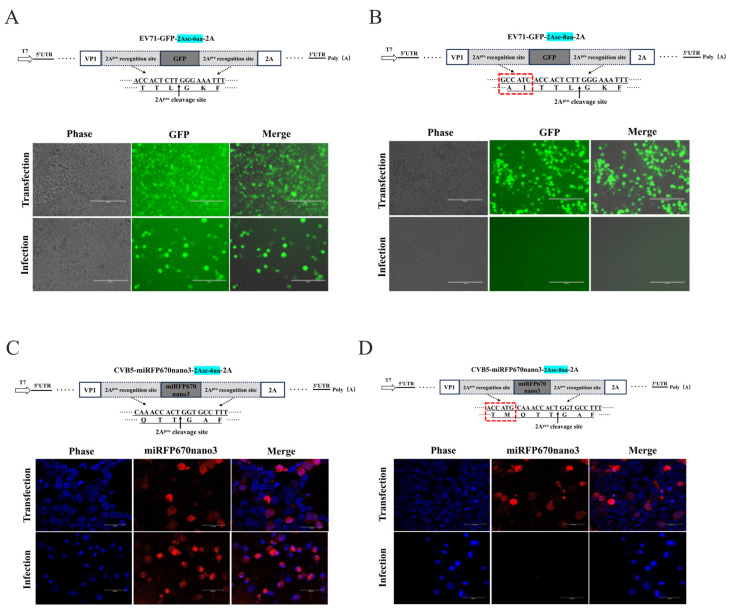
Effect of 2A^pro^ recognition consensus sequence length and integrity on recombinant reporter virus rescue. (**A**) pcDNA3.1-EV71-GFP-2Asc-6aa-2A and (**C**) pcDNA3.1-CVB5-miRFP670nano3-2Asc-6aa-2A infectious clones employ the basic 2A^pro^ recognition sequence (6 aa). (**B**) pcDNA3.1-EV71-GFP-2Asc-8aa-2A and (**D**) pcDNA3.1-CVB5-miRFP670nano3-2Asc-8aa-2A infectious clones employ the extended 2A^pro^ recognition sequence (8 aa). (**A**,**B**) were captured by inverted fluorescence microscopy; scale bar = 200 μm. (**C**,**D**) were captured by laser confocal microscopy; blue indicates DAPI-stained nuclei; scale bar = 40 μm. The extended 2 amino acids in the recognition sequence are highlighted by red boxes, and arrows indicate the cleavage site by viral protease 2A.

Theoretically, substrates with the full-length 2A protease recognition sequences should facilitate 2A^pro^ recognition and cleavage. Based on this hypothesis, to further optimize the protease recognition consensus sequence usage, we modified the cleavage strategy at this position by supplementing the P5 and P4 amino acid residues upstream of the minimal recognition sequence. Specifically, for the EV-A71, we added alanine (A) and isoleucine (I) to restore the complete AITTL↓GKF (8 aa) recognition sequences (Figure 7A,B). Surprisingly, at the VP1/2A site, extending the 2A consensus sequence from TTL↓G to AITTL↓G in EV71-GFP-2A abolished the rescue (Figure 7B). Similarly, extending the CVB5 sequence from QTT↓GAF to TMQTT↓GAF in pcDNA3.1-CVB5-miRFP670nano3-2A prevented virus recovery (Figure 7C,D). Notably, clones with the extended recognition sequence (8 aa) still showed robust fluorescence after the transfection—demonstrating the unimpaired viral genome transcription and translation (Figure 7B,D). These findings indicate that, at the VP1/2A cleavage site, the pursuit of relative completeness in the 2A^pro^ recognition sequence not only failed to enhance the packaging efficiency but completely abrogated the reporter virus rescue. This suggests that increasing the conservative sequence length likely induced local steric hindrance or conformational alterations in genomic RNA, thereby interfering with proper virion assembly.

### 3.7. Reporter Viruses with High Performance and Genetic Stability Carrying Novel Reporters Inserted at the VP1/2A Site

**Figure 8:**
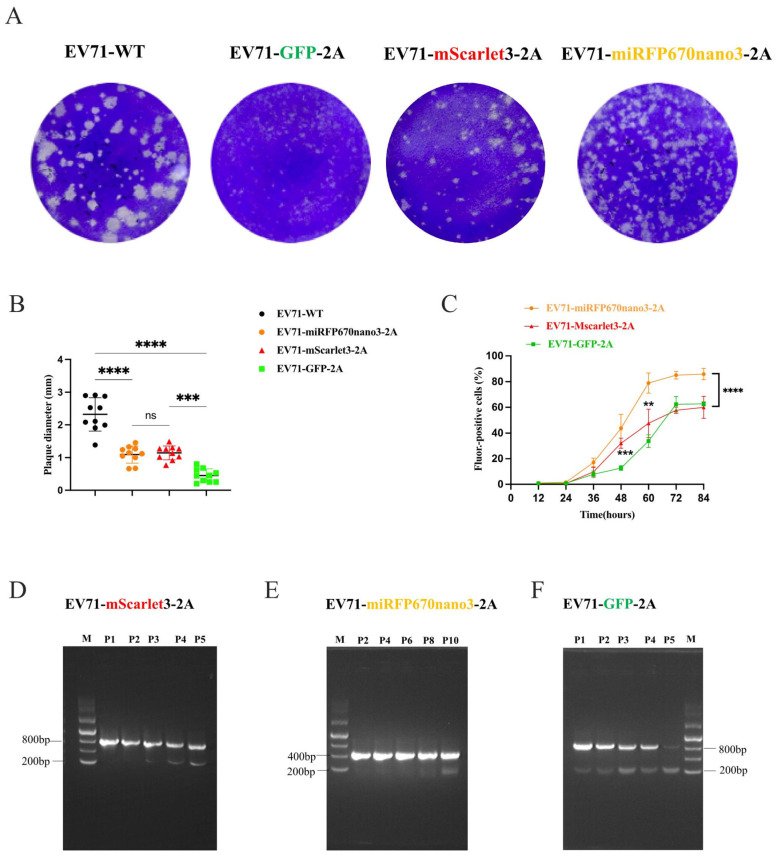
Characterization of novel EV-A71 reporter viruses integrated at the VP1/2A site. (**A**) Plaque assays of wild type EV-A71, EV71-GFP-2A, EV71-mScarlet3-2A, and EV71-miRFP670nano3-2A reporter viruses. (**B**) Plaque diameter quantification using Adobe Photoshop v.2021; 10 well-defined plaques per group were randomly selected for statistical analysis. Unpaired Student’s *t*-test, *** *p* < 0.001, **** *p* < 0.0001; and “ns” indicates no significant difference. (**C**) Growth curves of the three reporter viruses; cells were infected at an MOI of 0.1 and harvested at 12–84 hpi for flow cytometric analysis. Data represent two independent experiments, each performed in triplicate; two-way ANOVA with multiple comparisons at 36–84 hpi with EV71-GFP-2A reporter as control, ** *p* < 0.01, *** *p* < 0.001, and **** *p* < 0.0001. (**D**–**F**) Genetic stability analysis of EV71-mScarlet3-2A (**D**), and EV71-miRFP670nano3-2A (**E**), EV71-GFP-2A (**F**). Reporter viruses were used to infect RD cells and serially passaged for up to 10 passages; cells from each passage were collected when 70–80% of the infected RD cells exhibited fluorescence. The total RNA was extracted and followed by RT-PCR. Upper bands represent reporter-containing viral genomes; lower bands represent reporter-deleted viral fragments.

Flow cytometric analysis demonstrated significantly higher rescue efficiency at the VP1/2A site compared to 5′-UTR/VP4 (Figure 5E and Figure 6F). We therefore characterized three EV71 reporter viruses (GFP, mScarlet3, miRFP670nano3) and two CVB5 reporter viruses (mScarlet3, miRFP670nano3) at the VP1/2A site. The plaque assays revealed that reporter gene insertion reduced the plaque diameter in a size-dependent manner (*p* < 0.05) (Figure 8A,B). Growth curve analysis showed that, at equivalent initial MOIs, EV71-miRFP670nano3-2A exhibited the fastest replication, followed by EV71-mScarlet3-2A, with EV71-GFP-2A showing the slowest kinetics—consistent with the plaque assay results (Figure 8C). These reporter viruses were also serially passaged for up to 10 passages to assess their genetic stabilities. RT-PCR and semi-quantitative PCR revealed differential genetic stability: EV71-GFP (756 bp) showed substantial loss by passage 5; EV71-mScarlet3 (687 bp) remained predominantly intact at this passage; and EV71-miRFP670nano3 (438 bp) demonstrated the highest stability, with reporter gene loss only being evident at passage 10 (Figure 8D–F). Thus, mScarlet3- and miRFP670nano3-based reporter viruses offer substantial practical advantages. Consistent with EV-A71, CVB5 reporter viruses formed smaller plaques than the wild-type virus; however, CVB5-miRFP670nano3-2A produced larger and more distinct plaques than CVB5-mScarlet3-2A (Figure S5A,B) and exhibited markedly faster replication kinetics (Figure S5C,D).

### 3.8. EV-A71 and CVB5 Reporter Viruses Utilized for Drug Evaluation

**Figure 9:**
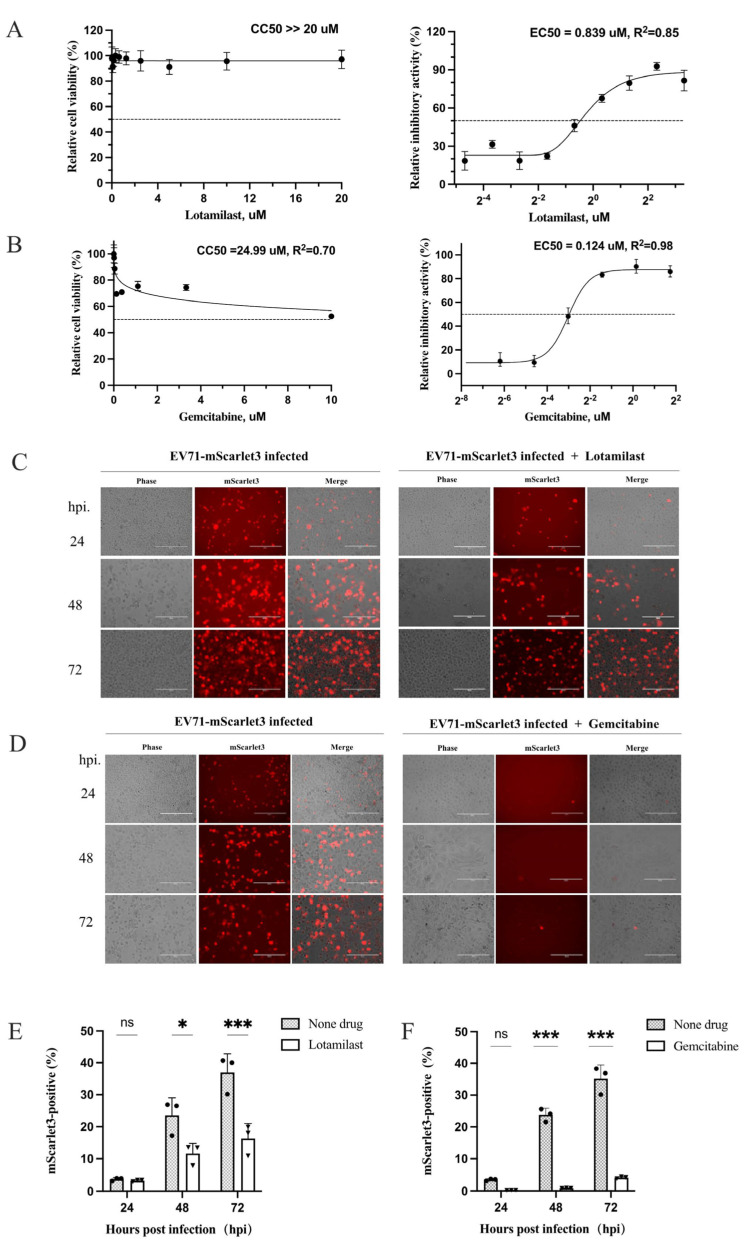
Candidate drugs with the activities for anti-EV71 and anti-CVB5. (**A**) EC_50_ and CC_50_ analysis of drug 3D2. RD cells were seeded in 96-well plates for 12 h. Then, the cells were treated by the drug, starting at 20 μM, and serially diluted in quintuple, and simultaneously infected with EV-A71 at an MOI of 0.5 for another 72 h. The cell viability was detected with CCK-8 kit for incubated 1.5 h. The values of CC_50_ and EC_50_ were counted by fitting the standard curves of dose–response. (**B**) EC_50_ and CC_50_ analysis of drug 5E4. The detection, as described in panel A. (**C**) Activity of lotamilast against EV71-mScarlet3-2A reporter virus. Cells were infected with the virus and treated or not by 3D2 at 1.0 μM. The reprehensive images from three independent experiments were captured at the indicated time points. (**D**) Activity of gemcitabine against EV71-mScarlet3-2A reporter virus. Detections were performed as in panel (**C**). (**E**,**F**) Quantification of fluorescent-positive cells by flow cytometric analysis after the reporter virus infection and treated or not by lotamilast (**E**) or gemcitabine (**F**) at the indicated time points. The cells were treated and collected from the panel (**C**,**D**), respectively. Data represent mean ± SD from three independent replicates; unpaired Student’s *t*-test, * *p* < 0.05, *** *p* < 0.001, and “ns”, not significant.

To validate the reporter viruses for antiviral screening, we selected two candidate compounds—lotamilast and gemcitabine—identified from an FDA-approved pediatric clinical drug library . We first determined the values of CC_50_, EC_50_, and the selectivity index (SI) for each compound, using cell viability as the readout. Lotamilast demonstrated excellent safety with no detectable cytotoxicity at 20 μM (CC_50_ >> 20 μM); combined with an EC_50_ of 0.839 μM, its estimated SI far exceeded 23.84 (Figure 9A). Gemcitabine exhibited exceptional potency with an EC_50_ of 0.124 μM and an SI of 201.5, indicating highly efficient target-specific inhibition (Figure 9B). We further evaluated the two compounds using our reporter viruses. The cells were infected with the EV71-mScarlet3-2A reporter virus and treated or not by lotamilast and gemcitabine. The results showed that the reporter viruses demonstrated clear advantages for evaluating lotamilast and gemcitabine antiviral efficacy: microscopy revealed markedly inferior inhibition by lotamilast compared to gemcitabine against EV-A71 (Figure 9C,D), with flow cytometric quantification confirming these observations (Figure 9E,F). CVB5-miRFP670nano3-2A reporter virus was employed to further evaluate the antiviral activity of gemcitabine. Flow cytometric analysis demonstrated that gemcitabine profoundly inhibited viral replication at 36 hpi: over 60% of the control cells were infected with miRFP670nano3-positive cells and the infection rate in the drug-treated group was reduced to less than 0.8% with virtually no CPE observed (Figure S6A,B).

### 3.9. Replication of EV-A71 and CVB5 Reporter Virus Inhibited in METTL3-KO Cells

**Figure 10:**
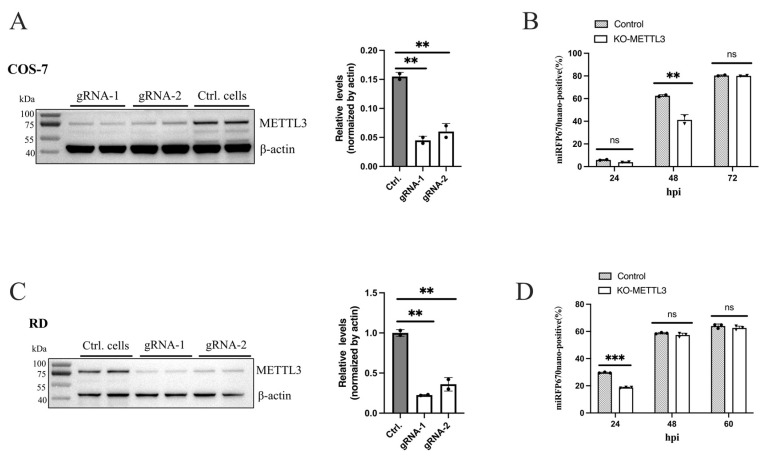
Replication of CVB5 and EV-A71 in cells inhibited by knockout of METTL3. (**A**) Generation of METTL3-knockout COS-7 cells. METTL3-knockout cells were constructed using the CRISPR-Cas9 system with gRNA-1 and gRNA-2, selected with puromycin, and assessed by immunoblotting with antibodies against METTL3 (**left**). Bands were quantified using ImageJ Version 1.51 and normalized to β-actin (**right**). (**B**) Knockout of METTL3 inhibited replication of reporter virus CVB5-miRFP670nano3-2A in COS-7 cells. METTL3-knockout cells generated with gRNA-1 were infected with reporter virus at MOI = 0.1 and analyzed by flow cytometry at indicated time points. (**C**) Generation of METTL3-knockout RD cells. Knockout RD cell pools were generated and assessed as in (**A**). (**D**) Knockout of METTL3 inhibited replication of reporter virus EV71-miRFP670nano3-2A in RD cells. METTL3-knockout RD cells generated with gRNA-1 were infected with reporter virus at MOI = 0.1 and analyzed as in (**B**). Data represent mean ± SD from three independent replicates; unpaired Student’s *t*-test, ** *p* < 0.01, *** *p* < 0.001, and “ns”, not significant..

Host factors play critical roles in viral infection. To further validate the utility of these reporter viruses for investigating host factor function, we selected METTL3 as a test case. N6-methyladenosine (m6A), the most abundant internal mRNA modification, is primarily catalyzed by a methyltransferase complex comprising METTL3 and associated factors. METTL3 has been identified as a key regulator of interferon responses by promoting the rapid turnover of interferon mRNAs, thereby facilitating viral propagation of influenza A virus and adenovirus [47]. METTL3-mediated m6A also positively regulates CVB3 replication and dynamically modifies Zika virus infection [48,49]. Here, we assessed the effects of METTL3-knockout on CVB5 and EV-A71 infection using novel reporter viruses. CVB5-miRFP670nano3-2A infection of METTL3-knockout COS7 cells revealed that at 24 hpi, both the control and knockout groups exhibited low baseline near-infrared fluorescence positivity (~5%). As infection progressed, the reporter virus entered rapid replication with markedly different efficiencies between groups. At 48 hpi, fluorescence positivity in the control cells rose to ~60%, whereas METTL3-knockout cells remained at ~40%, representing significant inhibition (*p* < 0.0001). However, this difference was abolished at 72 hpi (Figure 10A,B). Similar results were observed with EV71-miRFP670nano3-2A infection of METTL3-knockout RD cells: EV-A71 replication was inhibited at early stages in knockout cells, with the difference disappearing after 48 hpi (Figure 10C,D). These findings demonstrate that the host factor METTL3 functions as a proviral factor during the rapid replication stage of CVB5 and EV-A71 infection.

## 4. Discussions

Enterovirus causes many diseases, especially in young children. Infectious cDNA clones of enterovirus and their derived reporter viruses are powerful tools for virological research and drug evaluation. In a previous study, we constructed an EV-A71 infectious clone and established an efficient and rapid virus-rescue system based on the cells stably expressing T7 polymerase [23]. On the basis of that system, here we successfully constructed a CVB5 infectious clone and rescued the virus (Figure 1). The rescued virus showed similar biological characteristics to the parental virus in the induction of distinct CPEs, formation of plaques, and the growth curve of RNA replication and virus multiplication (Figure 2). The successful construction of the infectious clone provides a convenient reverse genetic tool for further studies on the structure and function of the CVB5 genome and the pathogenesis of the virus. Given that enterovirus genomes are comparable in size and organization, the streamlined strategy employed here—one-step PCR amplification, seamless cloning, and single-plasmid transfection into cells stably expressing T7 polymerase—should be readily applicable to the construction of infectious clones and rescue of other enteroviruses.

For reporter virus construction, the selection of appropriate insertion sites for reporter genes is critical for enterovirus reporter virus construction, as different sites exhibit varying tolerance for foreign sequences. In this study, a systematic screening of five key genomic positions in EV-A71 and CVB5 revealed that only the VP1/2A and 5′-UTR/VP4 sites tolerated GFP insertion and yielded viable EV-A71 reporter viruses (Figure 3, Figure 4 and Figure 6). Insertions within the P2 and P3 coding regions or upstream of the 3′-UTR consistently failed. These findings indicate that enteroviruses are highly sensitive to alterations in genomic spatial conformation; foreign gene introduction likely caused a deleterious steric hindrance and severely disrupted viral progeny assembly, rather than inhibiting viral translation, given that reporter expression was observed following the transfection of all clones. Among the sites tested, VP1/2A proved most effective for GFP-based reporter virus rescue.

For the 5′-UTR/VP4 site, previous studies have reported successful rescue of CVB5 reporter viruses carrying NanoLuc (Nluc) using 3C protease recognition sequences [30]. However, our attempts to employ 3C protease-mediated GFP release at this position—constructing pcDNA3.1-EV71-GFP-VP4 and pcDNA3.1-CVB5-GFP—failed for both EV-A71 and CVB5 (Figure 3 and Figure 4). In contrast, utilizing 2A protease at this site enabled successful rescue of EV71-GFP, demonstrating that 2A protease sites are superior to 3C sites at this genomic position (Figure 6). The reporter gene size may contribute to this discrepancy, as GFP (~27 kDa) is substantially larger than NanoLuc (~19 kDa). The increased bulk of GFP likely imposes greater spatial constraints on genome–capsid interactions, thereby interfering with virion assembly. Indeed, substitution with smaller reporter genes not only improved EV-A71 rescue efficiency using 2A protease sites, but also enabled the successful generation of CVB5-miRFP670nano3 reporter viruses (Figure 6 and Figure S4).

At the VP1/2A site, utilizing the endogenous 2A protease recognition sequence, we observed an unexpected result. To achieve the relative completeness of cleavage sequences, we attempted to extend the 2A protease recognition sequence at the C-terminal of GFP from the minimal recognition site (TTL↓GKF) to the more complete consensus sequence (AITTL↓GKF). Surprisingly, this modification completely abolished the reporter virus rescue (Figure 7). This finding suggests that the conserved recognition sequence length, rather than “completeness,” is critical for viral rescue and packaging. However, given that GFP expression was largely unaffected, the failure more likely stems from RNA structural alterations induced by the six additional nucleotides, disrupting genome packaging and preventing viral rescue—a phenomenon that is worthy of further investigation.

The VP1/2A site demonstrated clear advantages for EV71-GFP reporter virus rescue; however, CVB5-GFP rescue remained unsuccessful at this site, suggesting that the recombinant genome exceeded its capsid packaging capacity. We therefore introduced smaller reporter genes—mScarlet3-H and the substantially smaller far-red protein miRFP670nano3—under the optimized strategy (VP1/2A site with the concise 2A cleavage site). As anticipated, the reduced reporter gene size proved decisive. This modification not only improved the EV-A71 rescue efficiency but also enabled the successful generation of CVB5 reporter viruses carrying mScarlet3-H and miRFP670nano3 (Figure S3). These results demonstrate that reporter gene size is a critical determinant of rescue success and indicate that different enteroviruses exhibit distinct tolerance limits for foreign genetic material, with CVB5 possessing a narrower genomic capacity than EV-A71.

Then, we characterized the rescued EV71 and CVB5 reporter viruses carrying the novel reporters inserted at the VP1/2A site (Figure 8 and Figure S5). Recombinant viruses formed slightly smaller plaques and exhibited delayed replication kinetics compared to wild-type strains; however, novel reporter viruses maintained a superior replication capacity and cell lysis ability, relative to GFP-based constructs. Genetic stability analysis confirmed that mScarlet3-H- and miRFP670nano3-based reporter viruses are more stable than GFP reporter viruses. These high-adaptation, low-background fluorescent reporter systems—particularly the far-red variants—will provide a robust foundation for investigating spatiotemporal infection dynamics in vivo and for high-throughput antiviral drug screening [50].

Traditional antiviral evaluation relies on cytopathic effect observation, cell viability assays, or quantitative measurement of viral progeny. Although some methods offer adequate throughput for large-scale primary screening, endpoint assays cannot directly and dynamically track viral replication in living cells. The EV71-mScarlet3-2A and CVB5-miRFP670nano3-2A reporter viruses constructed in this study effectively address this limitation. However, we also identified the constraints of multimode plate reader-based microplate fluorescence quantification due to instrumental performance limitations. To overcome this, we combined microscopic imaging with flow cytometric quantification. Flow cytometry enables the discrimination of infected cells at the single-cell level, clearly revealing the dynamic characteristics of the compounds lotamilast and gemcitabine in controlling viral proliferation across different infection time points (Figure 9).

From a clinical pediatric drug library, we identified gemcitabine as a high-performance antiviral candidate. Gemcitabine exhibited an EC_50_ of 0.124 μM against EV-A71, a CC_50_ of 24.99 μM, and an exceptional selectivity index (SI) of 201.5 (Figure 9), indicating substantial clinical translational value. Cross-validation using novel reporter viruses demonstrated that gemcitabine not only blocked EV71-mScarlet3-2A replication but also significantly inhibited CVB5-miRFP670nano3-2A proliferation (Figure 9 and Figure S6). Given that EV-A71 and CVB5 belong to the enterovirus species A and B, respectively, this potent cross-species inhibitory activity suggests that gemcitabine likely targets the conserved viral replication factors, or alternatively interferes with host factors that are essential for enteroviral replication. Gemcitabine is a pyrimidine nucleoside analog antimetabolite and an antineoplastic agent, which inhibits DNA synthesis and repair, resulting in autophagy and apoptosis [51]. Further investigations are needed to clarify the molecular mechanism of gemcitabine against enteroviruses.

METTL3-mediated m6A regulated host IFN responses, thereby modifying many viral propagations, including influenza A virus, adenovirus, Zika virus, and CVB3 [47,48,49]. Using the CVB5 and EV-A71 reporter viruses, we also demonstrated that METTL3 functions as a proviral factor promoting CVB5 and EV-A71 replication, further exemplifying the utility of these novel reporter systems. However, we did not further assess the effects of METTL3 on viral infection in animal models. As reported, utilization of the compact far-red fluorescent protein miRFP670nano3 would offer substantial advantages over other reporters for monitoring viral replication in vivo [50]. Notably, although these novel reporter viruses significantly improve the rescue efficiency and genetic stability compared with traditional GFP reporter viruses, several technical limitations remain. The far-red fluorescence of miRFP670nano3 requires confocal microscopy or flow cytometry for optimal detection, as it is not reliably visualized by standard fluorescence microscopy. Similarly, while mScarlet3-H exhibits strong, stable fluorescence with minimal photobleaching, the accurate quantification of the fluorescence intensity remains challenging due to the limited sensitivity of multifunctional microplate readers.

Conclusively, in this study, we rapidly constructed a novel infectious cDNA clone of CVB5 using similar methods to those used for EV-A71. Through the systematic construction and optimization of EV-A71 and CVB5 reporter viruses, we successfully generated two panels of reporter viruses with high titers and relatively stable genetic inheritance carrying the two fluorescence proteins mScarlet3-H and miRFP670nano3, which showed significant advantages over the other canonical fluorescence proteins. The reporter gene size, genomic insertion site, and protease recognition sites for reporter cleavage are crucial parameters for the construction of a reporter virus. The reporter viruses could be conveniently utilized for drug evaluation and basic virology research.

## Data Availability

The data that support the findings of this study are presented and supplemented in the supplemental data files. The detailed methods are available from the corresponding author upon reasonable request.

## Supplementary Materials

The following supporting information can be downloaded at: https://www.mdpi.com/article/10.3390/v18050514/s1. Figure S1: Strategies for inserted GFP in viral genome and flowchart of screening of productive reporter virus, related to Figure 4; Figure S2: Construction and rescue evaluation of CVB5 reporter viruses with GFP-tag at distinct genomic sites, related to Figure 4; Figure S3: Novel fluorescence proteins exhibited advantages over GFP in the rescue of CVB-5 reporter virus, related Figure 5; Figure S4: Viral protease cleavage strategy for reporter release significantly impacted the efficiency of rescuing CVB5 reporter virus, related to Figure 6; Figure S5: Characterization of CVB5 reporter viruses with different reporters at the VP1/2A site, related to Figure 8; Figure S6: Evaluation of 5E4 antiviral activity against CVB5-miRFP670nano3-2A, related to Figure 9.
