# Supplementary material for: Rapid Construction and Characterization of Infectious cDNA Clones and Reporter Viruses of Enteroviruses, Including Enterovirus A71 and Coxsackievirus B5, with Systematic Identification of Critical Determinants for Successful Reporter Virus Generation
Source: Viruses. 2026 Apr 29;18(5):514. doi: 10.3390/v18050514 (PMC13211447; doi:10.3390/v18050514)
Supplement: Supplementary file 1 [file viruses-18-00514-s001.zip › viruses-4259410-supplementary.pdf]

Supplemental Figure legends

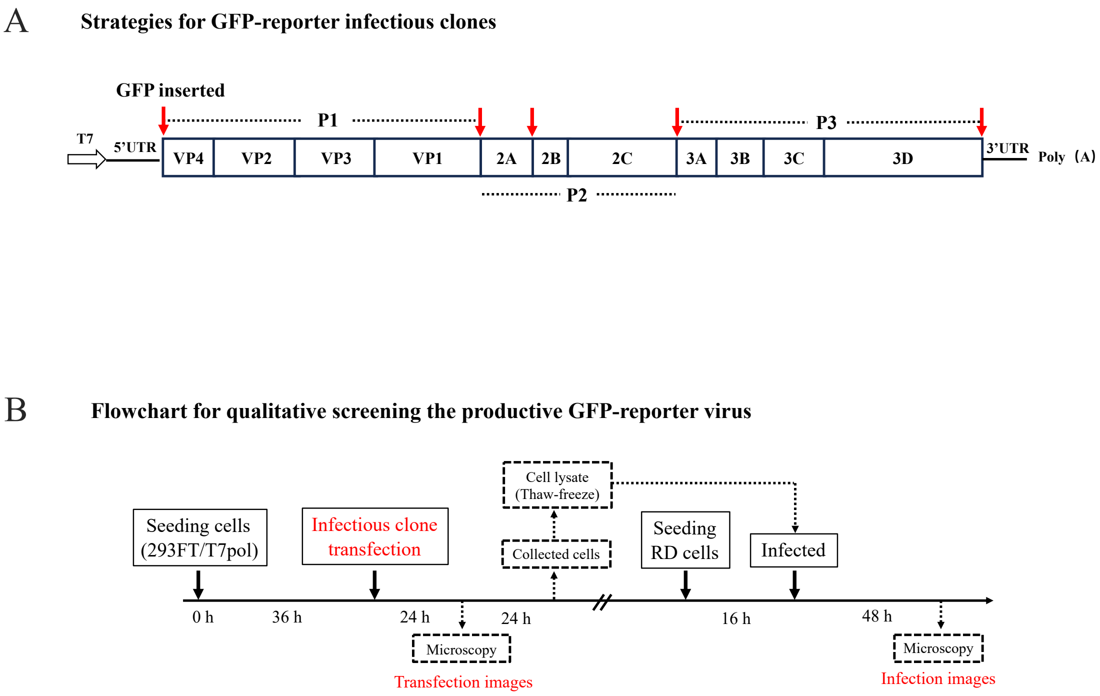

Fig. S1 Strategies for inserted GFP in viral genome and flowchart of screening of productive reporter virus, related to Fig. 4.

(A) Schematic illustrating the construction design and genomic sites screened for GFP insertion in the EV-A71 genome: 5′-UTR/VP4, VP1/2A, 2A/2B, 2C/3A, and 3D/3′-UTR. (B) Flowchart of productive reporter virus screening. Recombinant infectious clones were transfected into 293FT-T7pol cells and examined by microscopy at 24 h post-transfection (hpt). Cell lysates were collected at 48 hpt and used to infect RD cells; images were captured at 48 h post-infection (hpi). Each infectious clone was evaluated through at least three independent cycles of transfection and infection.

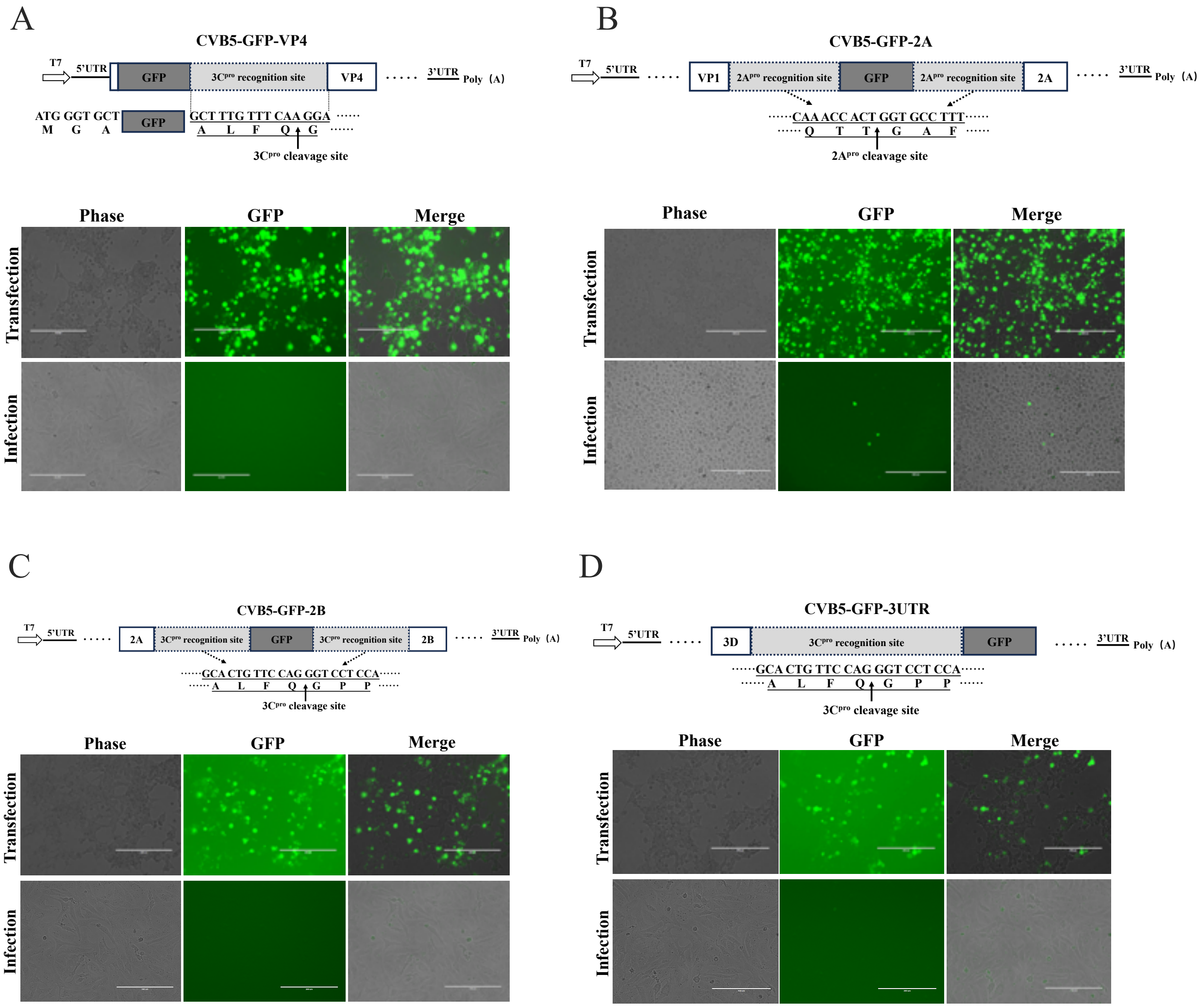

Fig. S2 Construction and rescue evaluation of CVB5 reporter viruses with GFP-tag at distinct genomic sites, related to Fig. 4.

(A–D) illustrate the construction design and rescue outcomes for GFP insertion at four candidate sites in the CVB5 genome: 5′-UTR/VP4 (A), VP1/2A (B), 2A/2B (C), and 3D/3′-UTR (D). Plasmids were transfected into 293FT-T7pol cells, and viral lysates were inoculated onto Vero cells. Scale bar: 400 µm for panel B (Infection); 200 µm for all others.

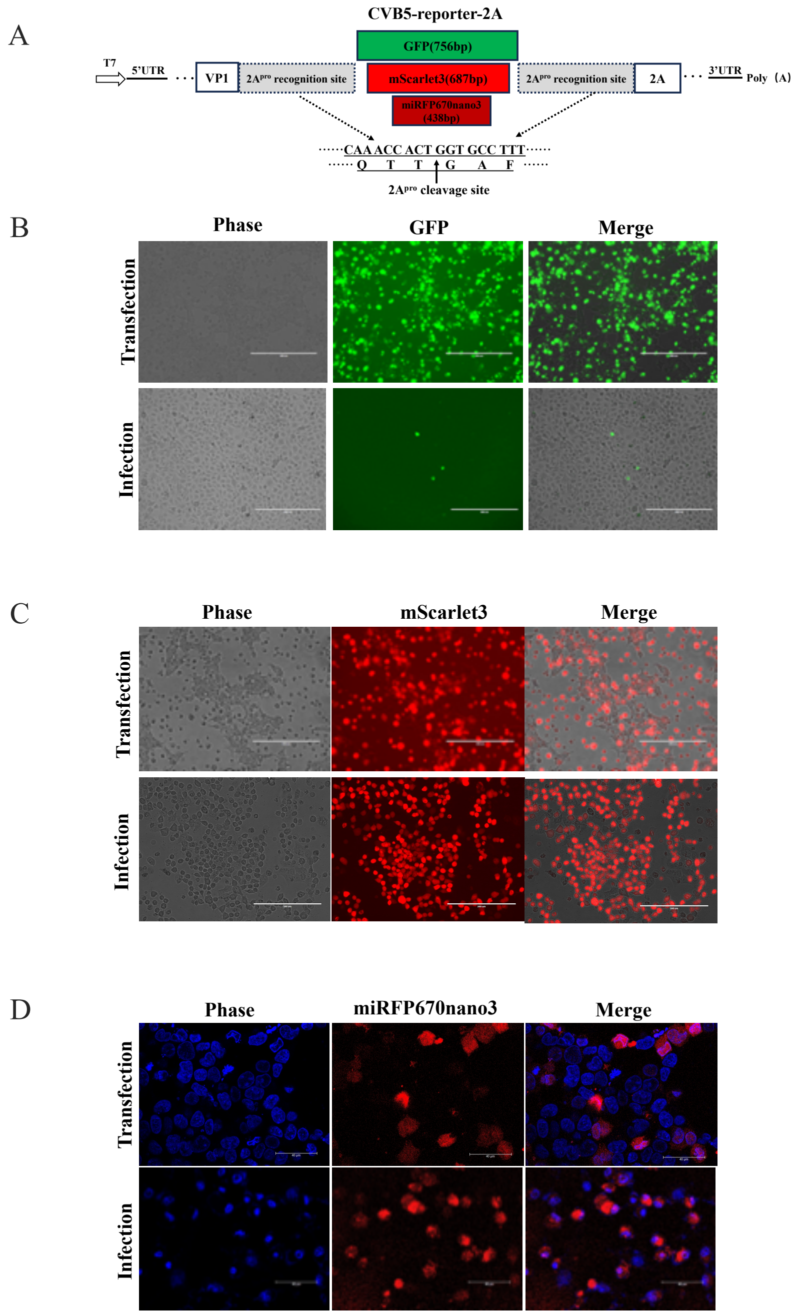

Fig. S3 Novel fluorescence proteins exhibited advantages over GFP in the rescue of CVB-5 reporter virus, related Fig. 5.

(A) Schematic of reporter virus construction strategy showing novel reporter gene insertion between VP1 and 2A in the CVB5 genome. (B, C) Rescue and infection validation of pcDNA3.1-CVB5-mScarlet3-2A (B) and pcDNA3.1-CVB5-miRFP670nano3-2A (C) infectious clones following transfection of 293FT-T7pol cells captured by fluorescent microcopy or confocal microscopy, respectively. Scale bars: 200 μm for (B); 40 μm for (C).

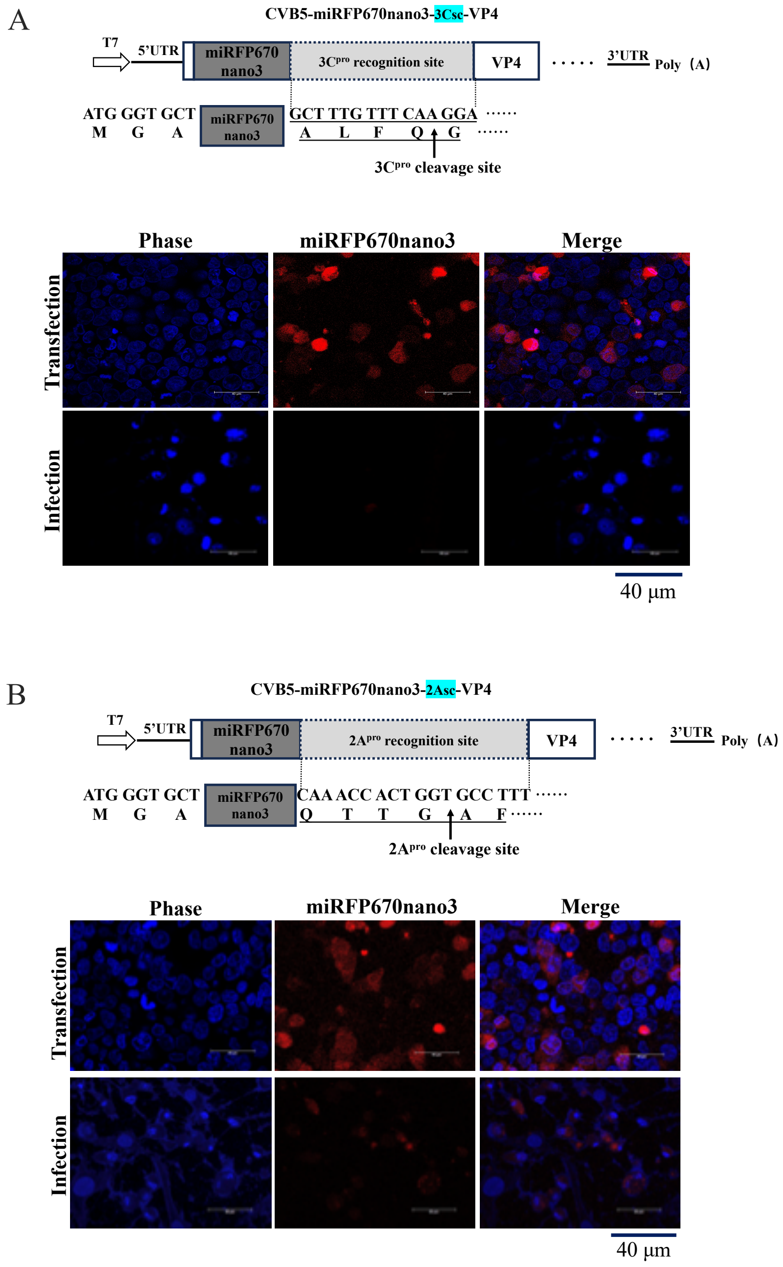

Fig. S4 Viral protease cleavage strategy for reporter release significantly impacted the efficiency of rescuing CVB5 reporter virus, related to Fig. 6. Optimization of protease cleavage strategy ($3C^{pro} \rightarrow 2A^{pro}$) for rescue of CVB5 reporter virus at the 5′-UTR/VP4 site. (A) pcDNA3.1-CVB5-miRFP670nano3-3Csc-VP4 infectious clone transfected into 293FT-T7pol cells and cell lysate used to infect RD cells. (B) Replacement with 2A protease recognition sequence, generating pcDNA3.1-CVB5-miRFP670-2Asc-VP4. Images were captured by confocal microscopy; blue indicates DAPI-stained nuclei. Scale bar at 40 μm.

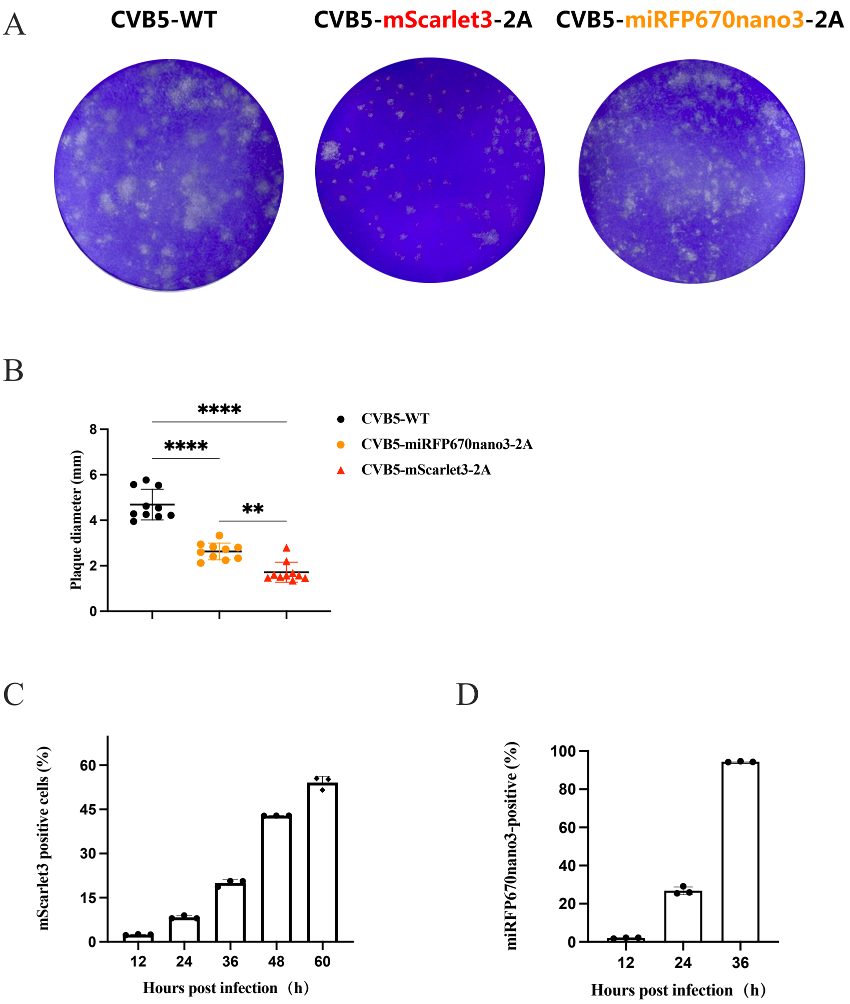

Fig. S5 Characterization of CVB5 reporter viruses with different reporters at the VP1/2A site, related to Fig.8. (A) Plaque assays of CVB5-WT, CVB5-mScarlet3-2A, and CVB5-miRFP670nano3-2A. (B) Plaque diameter quantification using Adobe Photoshop v.2021; 10 well-defined plaques per group were randomly selected for statistical analysis. Unpaired *t*-test, $**P < 0.01$, $****P < 0.0001$. (C, D) Growth of CVB5-mScarlet3-2A (C) and CVB5-miRFP670nano3-2A reporter virus (D); HeLa cells were infected with reporter virus at an MOI of 0.1 and harvested at indicated time points for flow cytometric analysis.

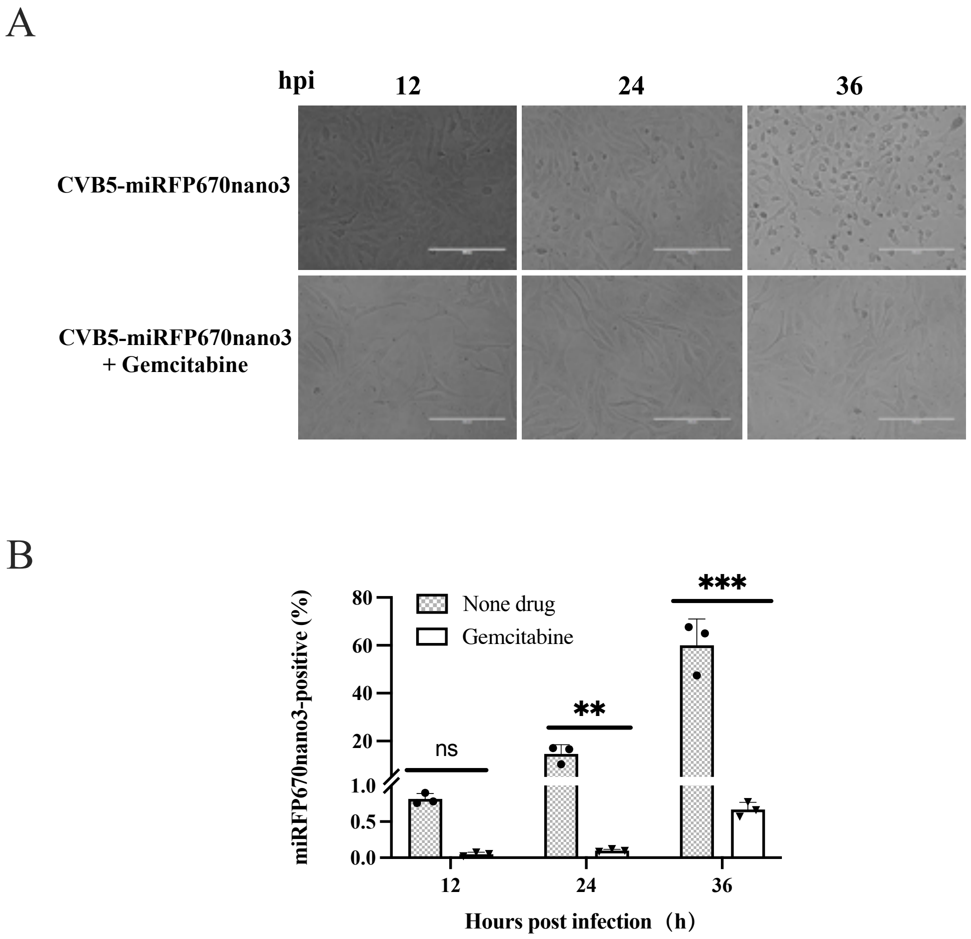

Fig. S6 Evaluation of 5E4 antiviral activity against CVB5-miRFP670nano3-2A.

(A) Cell morphology following reporter virus infection with or without 5E4 treatment. Vero cells were seeded in 24-well plates and infected with CVB5-miRFP670nano3-2A (MOI = 0.5) in the presence of 5E4 (1.0 μM); scale bar = 200 μm. (B) Quantification of miRFP670nano3-positive cells by flow cytometry. Data represent mean ± SD (n = 3); statistical analysis by the unpaired *t*-test. “ns” indicates no significant difference; $^{**}P < 0.01$; $^{***}P < 0.001$.
